# Proteolytic cleavages in the extracellular domain of receptor tyrosine kinases by membrane-associated serine proteases

**DOI:** 10.18632/oncotarget.17009

**Published:** 2017-04-10

**Authors:** Li-Mei Chen, Karl X. Chai

**Affiliations:** ^1^ Burnett School of Biomedical Sciences, Division of Cancer Research, University of Central Florida College of Medicine, Orlando, FL 32816-2364, USA

**Keywords:** receptor tyrosine kinase, matriptase, prostasin, Herceptin, breast cancer

## Abstract

The epithelial extracellular membrane-associated serine proteases matriptase, hepsin, and prostasin are proteolytic modifying enzymes of the extracellular domain (ECD) of the epidermal growth factor receptor (EGFR). Matriptase also cleaves the ECD of the vascular endothelial growth factor receptor 2 (VEGFR2) and the angiopoietin receptor Tie2. In this study we tested the hypothesis that these serine proteases may cleave the ECD of additional receptor tyrosine kinases (RTKs). We co-expressed the proteases in an epithelial cell line with Her2, Her3, Her4, insulin receptor (INSR), insulin-like growth factor I receptor (IGF-1R), the platelet-derived growth factor receptors (PDGFRs) α and β, or nerve growth factor receptor A (TrkA). Western blot analysis was performed to detect the carboxyl-terminal fragments (CTFs) of the RTKs. Matriptase and hepsin were found to cleave the ECD of all RTKs tested, while TMPRSS6/matriptase-2 cleaves the ECD of Her4, INSR, and PDGFR α and β. Prostasin was able to cleave the ECD of Her3 and PDGFRα. Matriptase cleaves phosphorylated Her2 at Arg558 and Arg599 and the Arg599 cleavage produces a CTF not recognized by the monoclonal antibody trastuzumab/Herceptin. Her2 cleavages by matriptase can be inhibited by the hepatocyte growth factor activator inhibitor 1 (HAI-1) in the MDA-MB-231 human breast cancer cells. Matriptase silencing in the Her2, matriptase, and HAI-1 triple-positive SKBR3 human breast cancer cells enhanced Her2 protein down-regulation induced by a sustained exposure to phorbol 12-myristate 13-acetate (PMA), which down-regulated matriptase protein. The novel Her2 cleavage and expression regulation mechanisms mediated by matriptase may have potential impacts in Her2-targeting therapies.

## INTRODUCTION

We have previously reported that the extracellular domain (ECD) of the epidermal growth factor receptor (EGFR, aka, Her1/ErbB1) is proteolytically modified by membrane-associated serine proteases, matriptase (aka, MT-SP1/epithin/ST14/PRSS14), prostasin (aka, CAP1/PRSS8), and hepsin (aka, TMPRSS1) in cultured epithelial cells [1, 2]. The amino-terminally truncated EGFR fragments produced by matriptase cleavages are tyrosine phosphorylated and activate the extracellular signal-regulated kinases (erk1/2) [1]. The amino-terminally truncated EGFR fragments produced by hepsin cleavages are also tyrosine phosphorylated but it is unclear what downstream signaling pathways are activated as a result [2]. Prostasin, upon its proteolytic activation, can enhance the EGFR ECD cleavages by matriptase, but had no effect on hepsin cleavages [1, 2].

Matriptase was reported to also cleave the ECD of the vascular endothelial growth factor receptor 2 (VEGFR2, aka, KDR), inactivating this RTK and its downstream signaling [3]. Matriptase was further reported to cleave the angiopoietin receptor Tie2 ECD to activate this RTK upon a membrane translocation mediated by protein kinase C (PKC), under sustained exposure to a high dose of phorbol 12-myristate 13-acetate (PMA) [4].

In this study, we tested the hypothesis that the membrane-associated extracellular serine proteases, matriptase, prostasin, and hepsin may cleave the ECD of additional RTKs. We investigated the protease-specific cleavages in the ECD of the following RTKs, the Her2/neu/ErbB2, Her3/ErbB3, Her4/ErbB4 of the Her/ErbB subfamily; the insulin receptor (INSR) and the insulin-like growth factor I receptor (IGF-1R) of the insulin receptor subfamily; the platelet-derived growth factor receptors (PDGFRs) α and β of the PDGFR subfamily; and nerve growth factor receptor A (TrkA) of the Trk receptor subfamily. Her2 cleavages by matriptase were investigated further in human breast cancer cells. Matriptase-specific cleavage sites in the Her2 ECD were determined and effects of these cleavages on a monoclonal antibody drug targeting the Her2 ECD, trastuzumab/Herceptin were studied. Matriptase was further implicated in Her2 expression regulation in response to sustained exposure to PMA, which also regulates matriptase expression.

## RESULTS

### Proteolytic modification of the Her/ErbB subfamily RTKs

**Figure 1:**
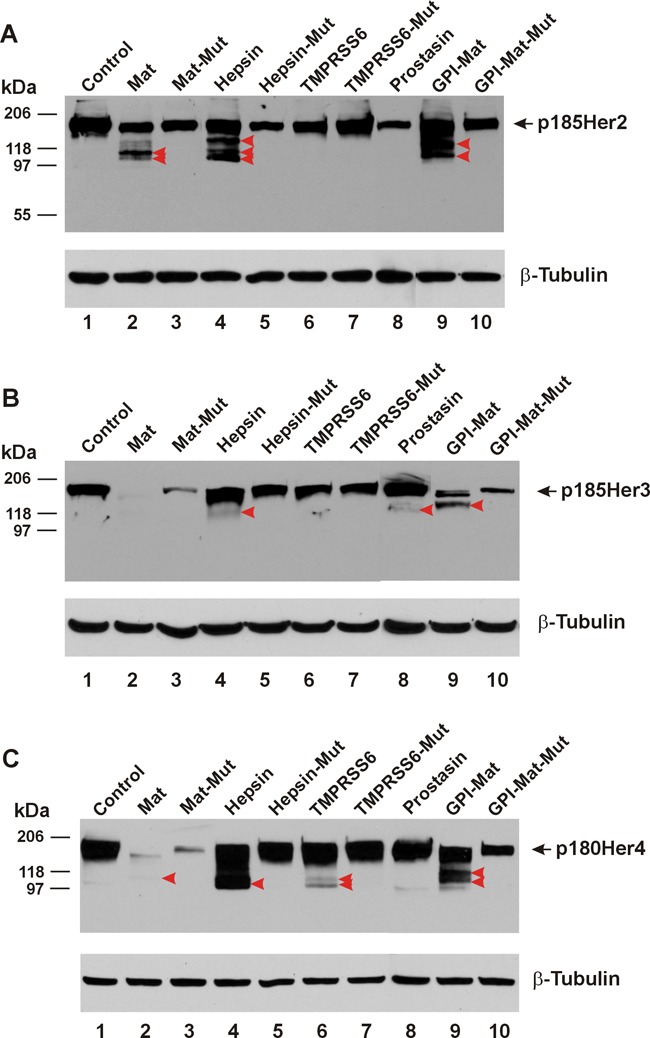
TTSPs are pan-ErbB proteolytic enzymes The cDNA coding for a carboxyl-terminally HA-tagged Her/ErbB receptor was co-transfected in HEK293 cells with an empty plasmid (pcDNA3, Invitrogen) (Control, Lane 1), or the cDNA coding for human matriptase (Mat, Lane 2), protease-dead matriptase (Mat-Mut, Lane 3), Hepsin (Lane 4), protease-dead hepsin (Hepsin-Mut, Lane 5), mouse TMPRSS6 (Lane 6), protease-dead mouse TMPRSS6 (TMPRSS6-Mut, Lane 7), human Prostasin (Lane 8), a GPI-anchored matriptase (GPI-Mat, Lane 9) and protease-dead GPI-anchored matriptase (GPI-Mat-Mut). Twenty micrograms of total cell lysate from each sample were resolved on SDS-PAGE and western-blotted with an anti-HA antibody or an anti-β-tubulin antibody. The full-length p185Her2 **(A)**, p185Her3 **(B)**, or p180Her4 **(C)** is indicated by the arrow, and the CTFs are indicated by the red arrowheads.

The type-II transmembrane serine proteases matriptase and hepsin and the GPI-anchored serine protease prostasin are all expressed as extracellular proteolytic enzymes, proteolytic cleavages on any receptor tyrosine kinase are therefore, expected to occur only in the receptor's ECD. Upon such cleavages with the truncations of the receptor from the amino terminus, detection via an antibody recognizing epitopes in the intracellular carboxyl terminal region will produce shorter fragments in a western blot. As shown in Figure 1A, Her2/neu/ErbB2 (HA-tagged) co-expressed with the human matriptase or hepsin was apparently cleaved to produce amino-terminally truncated Her2 carboxyl-terminal fragments (CTFs) that were picked up by the HA antibody used in the western blot. Major fragments migrating faster than the full-length receptor, p185Her2 as indicated by the black arrowhead were marked by the red arrowheads, in Lanes 2, 4, and 9 to show the apparent proteolytic cleavages by matriptase (Mat), Hepsin, and a GPI-anchored matriptase (GPI-Mat). No cleavages were observed when Her2 was co-expressed with the protease-dead mutant of matriptase (Mat-Mut) or hepsin (Hepsin-Mut), an indication that the proteolytic activities of matriptase or hepsin were required to generate the Her2 CTFs.

Prostasin however, could not cleave Her2 in this co-expression experiment as no Her2 CTFs were detected in Lane 8. We had previously shown that the full-length GPI-anchored prostasin expressed in this cell line was present on the plasma membrane as an active protease [9]. We can rule out membrane localization by the GPI anchor being a factor contributing to prostasin's inability to cleave the Her2 ECD because the GPI-anchored matriptase readily cleaved Her2 (Lane 9), whereas the GPI-anchored protease-dead mutant matriptase (GPI-Mat-Mut) did not.

To determine if other members of the type-II transmembrane extracellular serine protease (TTSP) family can also cleave the Her/ErbB subfamily RTKs, we co-expressed the mouse TMPRSS6 serine protease, aka matriptase-2 or its protease-dead mutant (TMPRSS6-Mut) with Her2. This TTSP however, was unable to cleave the Her2 ECD (Lane 6, Figure 1A), whereas the enzyme expressed from the same cDNA plasmid was capable of cleaving its target substrate hemojuvelin [5]. The cDNA plasmids coding for the proteases used in this study have been previously used in published studies [1, 2, 5] and the proper expression of the proteases was confirmed by using antibodies specific for the proteases (matriptase, hepsin, and prostasin) or a fused tag (FLAG, for TMPRSS6) (data not shown).

We have shown previously [1, 2] that matriptase and hepsin cleaved the Her1/ErbB1/EGFR ECD whereas the extent of sequence similarity in the ECD is very high in the Her/ErbB subfamily RTKs. In the ECD Her2 shares 44% amino acid identity with EGFR and the amino acid identity shared between the ECD of Her3 and that of EGFR is 45.9% in a 610-residue alignment region. The amino acid identity shared between the ECD of Her4 and that of EGFR is 48.3% in a 611-residue alignment region. We asked if the TTSPs are pan-ErbB ECD modifying enzymes in the next set of experiments. We show in Figure 1B that co-expression of matriptase, hepsin, or the GPI-anchored matriptase could produce Her3 ECD cleavages whereas TMPRSS6 failed to do so. Prostasin produced a weakly detectable Her3 CTF. As for Her4, matriptase, hepsin and GPI-anchored matriptase were shown to cleave its ECD, while TMPRSS6 had a weak activity toward this Her/ErbB subfamily RTK (Figure 1C). A longer exposure of the western blots provided a clearer identification of the Her3 and Her4 CTFs generated by matriptase (Supplementary Figure 1). For Her2, Her3, and Her4, co-expression with some proteases or the protease-dead mutant forms was associated with a reduction of the intact receptor level without a clearly identifiable cleavage product. This phenomenon was seen with Her2 (comparing Lanes 3, 5, 6, 8, and 10 to Lane 1), but was more pronounced when the protease-dead mutant matriptase (transmembrane or GPI-anchored) was co-expressed with Her3 (Figure 1B) or Her4 (Figure 1C). With the mechanism of the reduction in quantity of the intact receptor by the protease-dead mutants remaining to be investigated, we are only identifying proteolytic enzymes of the ErbB receptors with a clear detection of the CTFs in these experiments.

### Proteolytic modification of other RTK subfamilies

Matriptase can cleave the ECD of RTKs in three different subfamilies, the Her/ErbB ([1] and this study), the VEGFR [3], and the TIE receptor [4]. We reckoned that the TTSPs may have an even broader substrate range among the RTK subfamilies. We selected the following RTKs for testing ECD cleavages by the serine proteases based on the receptor involvement in epithelial cancer pathogenesis and the immediate availability of the cDNA.

**Figure 2:**
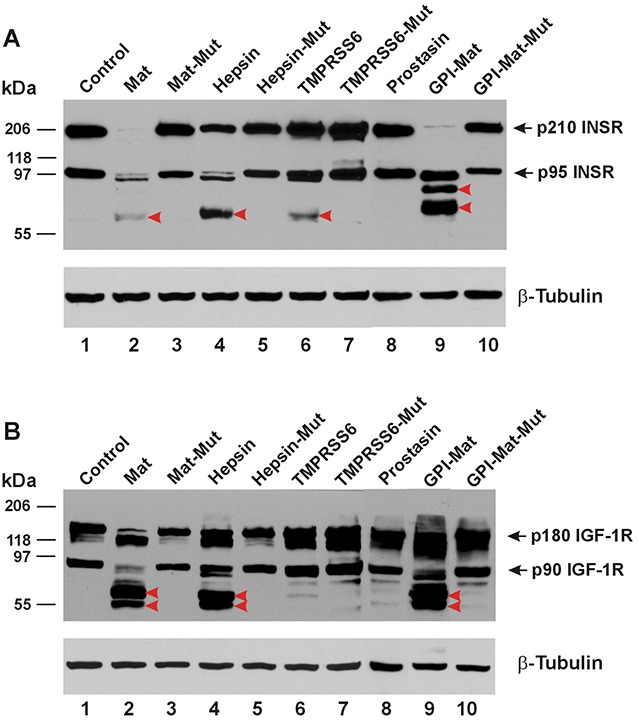
TTSPs proteolytically modify INSR and IGF-1R The cDNA coding for a carboxyl-terminally HA-tagged receptor was co-transfected in HEK293 cells with an empty plasmid (pcDNA3, Invitrogen) (Control, Lane 1), or the cDNA coding for human matriptase (Mat, Lane 2), protease-dead matriptase (Mat-Mut, Lane 3), Hepsin (Lane 4), protease-dead hepsin (Hepsin-Mut, Lane 5), mouse TMPRSS6 (Lane 6), protease-dead mouse TMPRSS6 (TMPRSS6-Mut, Lane 7), human Prostasin (Lane 8), a GPI-anchored matriptase (GPI-Mat, Lane 9) and protease-dead GPI-anchored matriptase (GPI-Mat-Mut). Twenty micrograms of total cell lysate from each sample were resolved on SDS-PAGE and western-blotted with an anti-HA antibody or an anti-β-tubulin antibody. The p210 INSR and p95 INSR **(A)**, or p180 IGF-1R and p90 IGF-1R **(B)** are indicated by the arrows, and the CTFs are indicated by the red arrowheads.

In Figure 2A, we show that the insulin receptor (INSR) can be cleaved in its ECD by matriptase, hepsin, the GPI-anchored matriptase, and also by TMPRSS6. Matriptase and the GPI-anchored matriptase produced apparently different cleavages in the insulin receptor ECD. In Figure 2B, we show the cleavages in the ECD of the insulin-like growth factor I receptor (IGF-1R) induced by matriptase, hepsin, and the GPI-anchored matriptase. But TMPRSS6 was unable to cleave the IGF-1R ECD.

**Figure 3:**
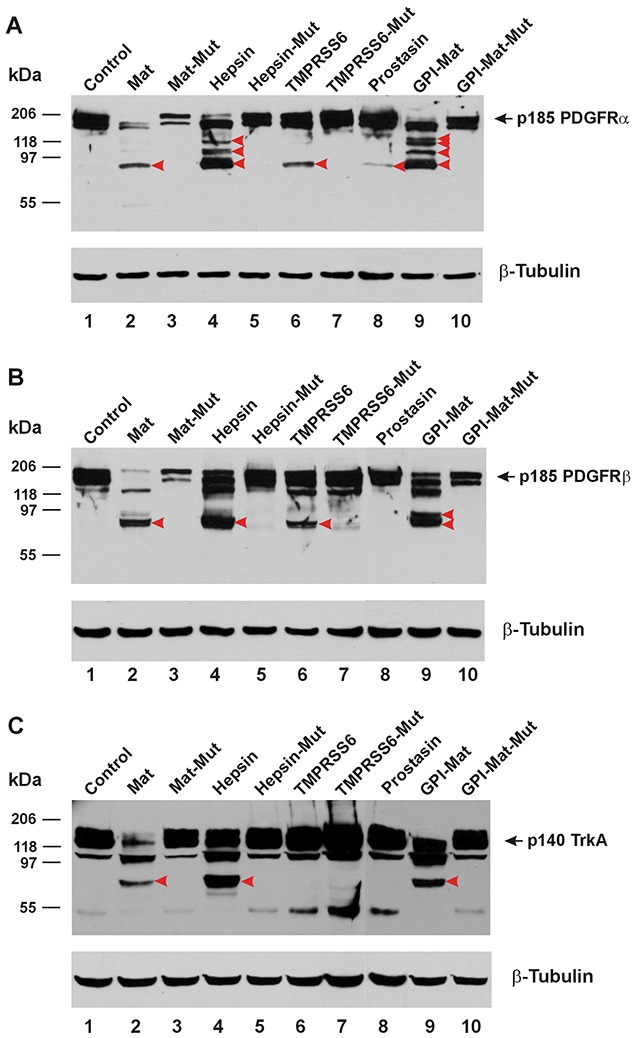
TTSPs proteolytically modify PDGFRs and TrkA The cDNA coding for a carboxyl-terminally HA-tagged receptor was co-transfected in HEK293 cells with an empty plasmid (pcDNA3, Invitrogen) (Control, Lane 1), or the cDNA coding for human matriptase (Mat, Lane 2), protease-dead matriptase (Mat-Mut, Lane 3), Hepsin (Lane 4), protease-dead hepsin (Hepsin-Mut, Lane 5), mouse TMPRSS6 (Lane 6), protease-dead mouse TMPRSS6 (TMPRSS6-Mut, Lane 7), human Prostasin (Lane 8), a GPI-anchored matriptase (GPI-Mat, Lane 9) and protease-dead GPI-anchored matriptase (GPI-Mat-Mut). Twenty micrograms of total cell lysate from each sample were resolved on SDS-PAGE and western-blotted with an anti-HA antibody or an anti-β-tubulin antibody. The p185 PDGFRα **(A)**, p185 PDGFRβ **(B)** or p140 TrkA **(C)** is indicated by the arrows, and the CTFs are indicated by the red arrowheads.

In Figure 3A, we show that the platelet-derived growth factor receptor α (PDGFRα) can be cleaved in its ECD by matriptase, hepsin, the GPI-anchored matriptase, and TMPRSS6. Prostasin also appeared to cleave the PDGFRα ECD producing a weakly detectable CTF similar in size to a CTF generated by the other proteases. In Figure 3B, we show the cleavages in the PDGFRβ ECD induced by matriptase, hepsin, the GPI-anchored matriptase, and TMPRSS6. Once again, co-expression of the protease-dead mutant matriptase resulted in quantity reduction of the intact PDGFRα or PDGFRβ without producing a clearly detectable CTF. In Figure 3C, we show that the nerve growth factor receptor TrkA can be cleaved in its ECD only by matriptase, hepsin, and the GPI-anchored matriptase, but not TMPRSS6.

### Arginine 558 and Arginine 599 of the Her2 ECD are cleavage sites for matriptase

**Figure 4:**
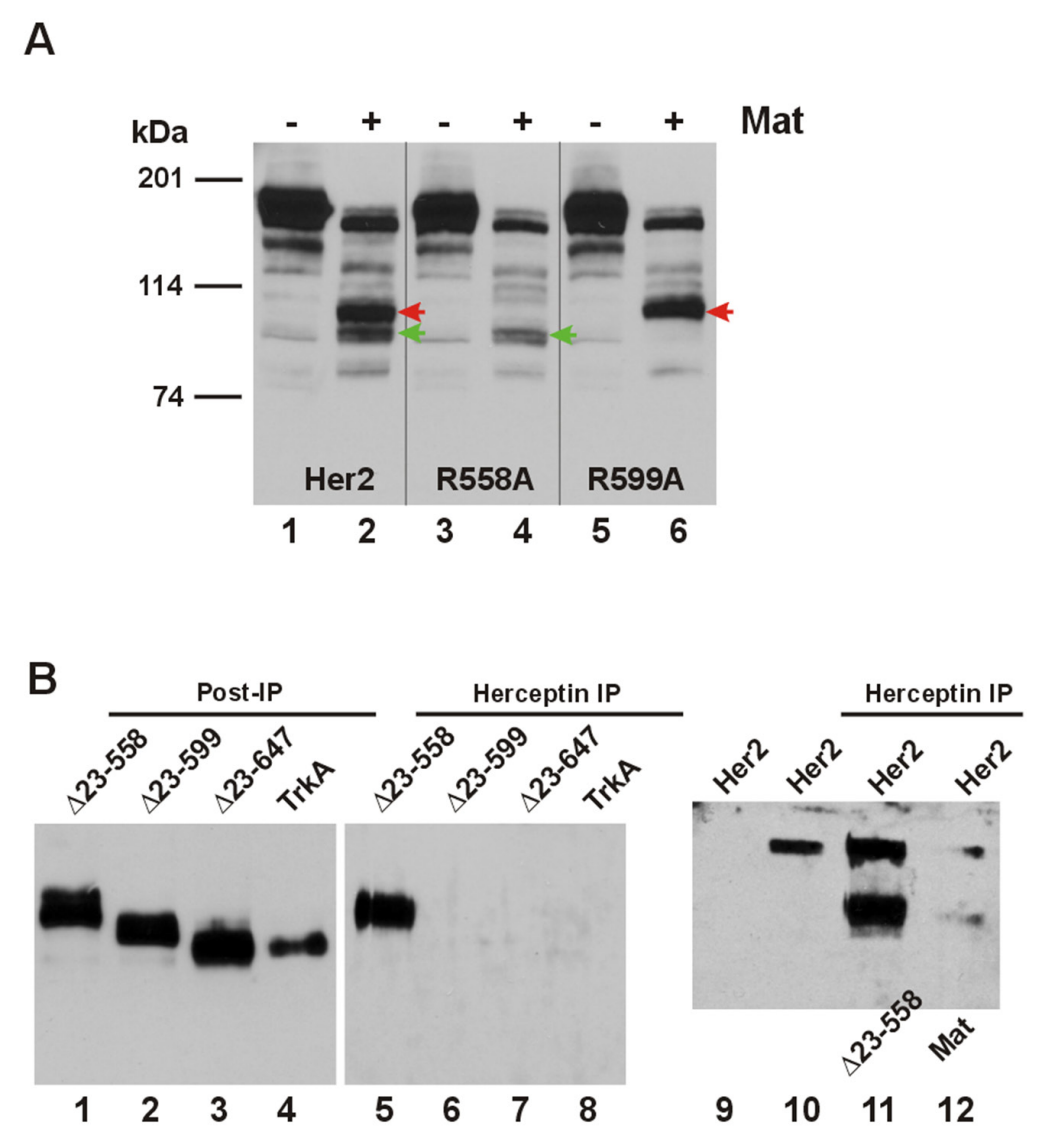
Matriptase-cleaved Her2 CTFs have differential interaction with Herceptin **(A)** The cDNA coding for a carboxyl-terminally HA-tagged wild-type Her2 or matriptase cleavage site mutant R558A or R599A was co-transfected in HEK293 cells with an empty plasmid (pcDNA3, Invitrogen) (Lane 1, 3, or 5), or the cDNA coding for human matriptase (Mat, Lane 2, 4, or 6). Twenty micrograms of total cell lysate from each sample were resolved on SDS-PAGE and western-blotted with an anti-HA antibody. The red arrowheads indicate the Her2 CTF resulting from matriptase cleavage at Arg558 and the green arrowheads indicate the Her2 CTF resulting from matriptase cleavage at Arg599. **(B)** The cDNA coding for amino-terminally truncated Her2s, Δ23-558, Δ23-599, or Δ23-647, or TrkA (all HA-tagged at the carboxyl terminus) was transfected in M17 human neuroblastoma cells, which were incubated with Herceptin for 24 hours. Immunoprecipitation (IP) and western blotting were performed as described in Materials and Methods. Samples from the IP were loaded in Lanes 5-8 and the post-IP samples were loaded in Lanes 1-4. The sample in Lane 9 was from cells not incubated with Herceptin, but the sample lysate was incubated with the agarose beads to serve as a negative control for the IP. The sample in Lane 11 was from cells co-transfected with the full-length Her2 and Δ23-558. The sample in Lane 12 was from cells co-transfected with the full-length Her2 and matriptase, as indicated. The sample in Lane 10 was from cells transfected with the full-length Her2 to serve as a positive control for the IP.

We have selected Her2 for a more detailed analysis of the ECD cleavages by matriptase because of our immediate interest in the ErbB receptors and this particular protease. The best natural and synthetic substrates of matriptase contain QAR at the P3-P1 positions [10, 11]. In the Her2 ECD we identified two candidate sites with AR at P2-P1 for matriptase cleavage, Arg558 and Arg599, to produce CTFs in the 100-120 kDa size range by estimate. When these two arginines were replaced with alanines, site-specific CTFs expected to be produced by matriptase were absent. In Figure 4A we identified the two major CTFs with red and green arrowheads (Lane 2). Replacing Arg558 with an Ala resulted in absence of CTF-His559 (cleaved at Arg558) identified by the red arrowhead when Her2-R558A was cleaved by matriptase (Lane 4) while replacing Arg599 resulted in absence of CTF-Cys600 (cleaved at Arg599) identified by the green arrowhead during matriptase cleavage of Her2-R599A (Lane 6). These data confirm Arg558 and Arg599 as two major sites for matriptase cleavage in the Her2 ECD.

The Arg558 and Arg599 matriptase cleavage sites fall within the epitope region (residues 557-603) recognized by the monoclonal antibody (mAb) drug Herceptin/trastuzumab [12]. We asked if Her2 CTFs generated by matriptase cleavages could still be recognized by the mAb. We deleted codons Thr23-Arg558 or Thr23-Arg599 of the Her2 cDNA to produce the cDNAs Δ23-558 and Δ23-599 coding for the two site-specific Her2 CTFs, and expressed these in the M17 neuroblastoma cells determined to be negative for both Her2 and matriptase expression (data not shown). Immunoprecipitation (IP) with the mAb Herceptin pulled down Her2 CTF-His559 expressed from Δ23-558 (Figure 4B, Lane 5), which should retain most of the mAb's recognition region; but not Her2 CTF-Cys600 expressed from Δ23-599 (Lane 6), which would have lost most of the mAb's recognition region. Another site-specific Her2 CTF expressed from the cDNA Δ23-647 with a deletion of codons Thr23-Arg647, Her2 CTF-Ala648 was made to model the α-secretase cleaved Her2 [13] and was also not pulled down by Herceptin (Lane 7). TrkA was used as a negative control for the IP experiment (Lane 8). In the presence of the full-length Her2, a Herceptin IP pulled down both the full-length receptor and the CTF expressed from Δ23-558 or the matriptase-cleaved (at Arg558) Her2 CTF-His559 when these two forms were co-transfected (Lane 11) or when the latter was generated via a matriptase cleavage (Lane 12).

### Her2 and matriptase expression in human breast cancer cells

**Figure 5:**
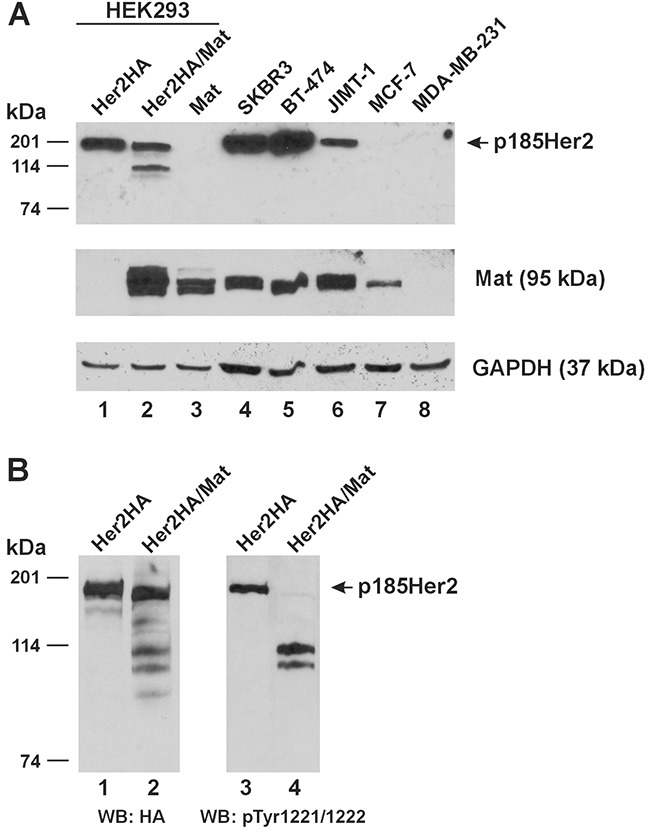
Relative expression of Her2 and matriptase in experimental cell lines and cleavage of phosphorylated Her2 by matriptase **(A)** An equal amount of total protein lysate (20 μg) from HEK293 cells transfected with Her2HA (Lane 1), Her2HA and matriptase (Mat, Lane 2), or matriptase (Mat, Lane 3), and the human breast cancer cells SKBR3 (Lane 4), BT-474 (Lane 5), JIMT-1 (Lane 6), MCF-7 (Lane 7), and MDA-MB-231 (Lane 8) was used for western blot analysis of Her2 (Top Panel) and matriptase (Middle Panel). A GAPDH western blot (Bottom Panel) was performed as a loading control. **(B)** HEK293 cells transfected with Her2HA (Lanes 1 and 3), or Her2HA and matriptase (Mat, Lanes 2 and 4) were analyzed for Her2(HA) (Left Panel) and phospho-Her2 (pTyr1221/1222) (Right Panel). WB: western blot.

We compared the Her2(HA) and matriptase levels expressed in the HEK293 cells for protease cleavage assays to the native Her2 and matriptase expressed in human breast cancer cells. In Figure 5A, we show that Her2(HA) (Lane 1) expressed in the HEK293 cells is between the levels of native Her2 in the JIMT-1 cells and that in the SKBR3 and BT-474 cells. The relative levels of Her2 detected in the SKBR3, BT-474, and JIMT-1 cells conformed to what had been reported previously in the literature [14]. Matriptase expressed alone in the HEK293 cells (Figure 5A, Lane 3) was at a level comparable to that in the SKBR3, BT-474, and JIMT-1 cells (Lanes 4-6) and more than that in the MCF-7 cells (Lane 7). The MDA-MB-231 cells (Lane 8) do not express matriptase, in agreement with a previous report [15].

### Matriptase cleaves tyrosine phosphorylated Her2

Whereas the matriptase-cleaved Her2 CTFs could readily be detected in the cell lysate when Her2 and matriptase were co-expressed in the HEK293 cells (Figures 1A, Lane 2), the p185Her2 does not appear to be completely cleaved by matriptase. More important, a question also remains at this point if matriptase cleaves Her2 presented on the plasma membrane where the receptor is physiologically relevant. Her2 over-expressed in breast cancer cells clusters on the plasma membrane and forms homodimers to activate cell signaling [16]. We further resolved the HEK293 cell samples shown in Lanes 1-2 of Figure 5A, and performed a western blot analysis using an antibody against the phosphorylated tyrosines at 1221/1222, shown in Figure 5B, Lanes 3-4. Her2 molecules phosphorylated at tyrosines 1221/1222 were completely cleaved by matriptase (Figure 5B, Lane 4). The remaining full-length Her2 in the cells co-expressing matriptase (Figure 5B, Lane 2) is likely unphosphorylated.

### Induction of matriptase in human breast cancer cells over-expressing Her2 results in site-specific ECD cleavage

The MDA-MB-231 human breast cancer cell line is Her2-negative/unamplified but a constitutive over-expression of Her2 in this cell line by genetic engineering produces a derivative cell line (231-H2N) regarded as clinically relevant [17]. We generated a similar cell line by transfecting the MDA-MB-231 with the pcDNA3-Her2(HA) plasmid and selecting for Her2(HA) over-expressing drug-resistant colonies, producing the 231H2. With this method, we also generated the 231R2A cell line, constitutively expressing a Her2(HA) double-mutant at Arg558Ala and Arg599Ala, resistant to matriptase cleavage. We then endowed the 231H2 and 231R2A cell lines with tetracycline (tet)-inducible matriptase expression via lentiviral transductions (Materials and Methods), producing the 231H2-TRM and 231R2A-TRM cell lines, respectively. We further engineered the 231H2-TRM cell line via lentiviral transduction to constitutively express the human hepatocyte growth factor activator inhibitor-1 (HAI-1), a reversible inhibitor of matriptase [18], producing the 231H2-TRM-H1 cell line.

**Figure 6:**
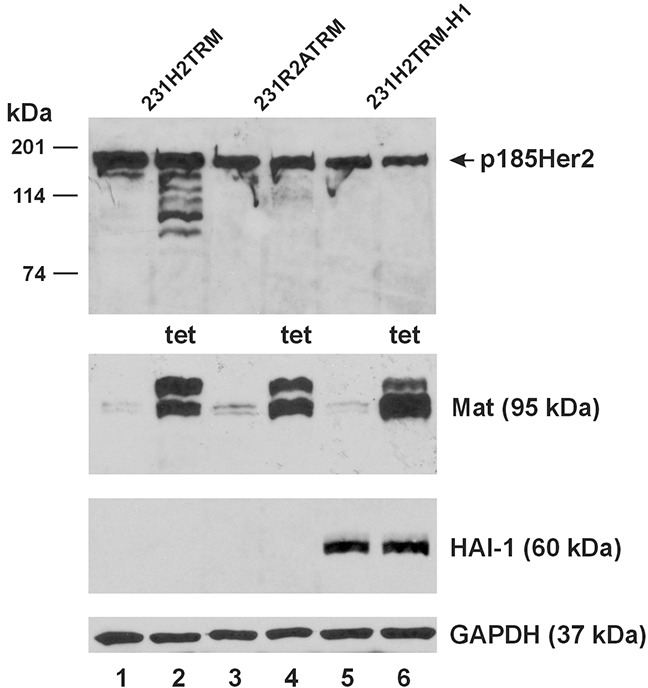
Matriptase cleaves stably expressed Her2 but not Her2 with mutations at the matriptase cleavage sites An equal amount of total protein lysate (40 μg) from the 231H2TRM (Lanes 1 and 2), 231R2ATRM (Lanes 3 and 4), and 231H2TRM-H1 (Lanes 5 and 6) cells was used for western blot analysis of Her2HA (Top Panel), matriptase (Mat, Second Panel from the Top), and HAI-1 (Third Panel from the Top). Cells represented by samples in Lane 2, 4, 6 were treated with 1 μg/ml tetracycline (tet) for 24 hours. A GAPDH western blot (Bottom Panel) was performed as a loading control.

Upon tet-induction in the 231H2-TRM, 231R2A-TRM, and the 231H2-TRM-H1 cells, matriptase was properly over-expressed, as shown in Figure 6, Lanes 2, 4 and 6; and the wild-type Her2(HA) was readily cleaved (Lane 2). The double-mutant Her2(HA) in the 231R2A-TRM cells was resistant to cleavage by the induced matriptase (Lane 4), as expected. Constitutive over-expression of HAI-1 in the 231H2-TRM-H1 cells (Lanes 5-6) completely inhibited the matriptase cleavage of the wild-type Her2(HA) (Lane 6).

### Matriptase gene knockout in Her2-amplified human breast cancer cells results in enhanced Her2 turnover when stimulated with PMA

**Figure 7:**
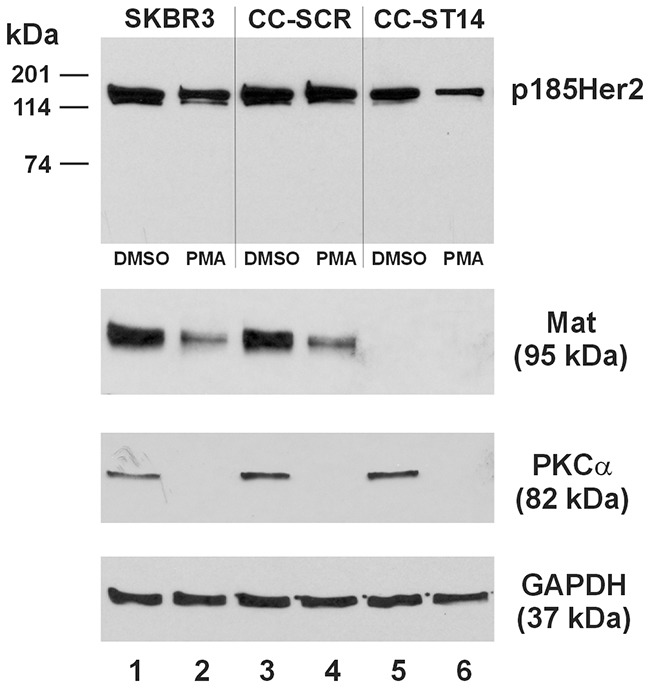
Matriptase knockout enhances PMA-induced Her2 turnover in SKBR3 human breast cancer cells An equal amount of total protein lysate (40 μg) from the SKBR3 (Lanes 1 and 2), SKBR3/CC-SCR (Lanes 3 and 4), and SKBR3/CC-ST14 (Lanes 5 and 6) cells was used for western blot analysis of Her2 (Top Panel), matriptase (Mat, Second Panel from the Top), and PKCα (Third Panel from the Top). Cells represented by samples in Lane 2, 4, and 6 were treated with 1 μM PMA for 16 hours, and those in Lanes 1, 3, and 5 with the vehicle control (DMSO). A GAPDH western blot (Bottom Panel) was performed as a loading control.

The MDA-MB-231 human breast cancer cell line is negative for both matriptase and HAI-1 [15], but is apparently capable of supporting the role of matriptase to cleave Her2 and the re-expressed matriptase is responsive to a re-expressed HAI-1 (Figure 6). The Her2-amplified SKBR3 human breast cancer cell line expresses both matriptase and HAI-1 [15]. We employed the sgRNA CRISPR/Cas9 technology via lentiviral transduction to establish a derivative cell line of the SKBR3, the SKBR3/CC-ST14, in which the matriptase (ST14) gene is knocked-out. In Figure 7, we show a successful matriptase gene knockout in the SKBR3/CC-ST14 cells, with no matriptase protein detected (Lanes 5 and 6), but the SKBR3/CC-SCR cell line generated with a scrambled sgRNA CRISPR/Cas9 lentivirus expresses the matriptase protein at a level (Lane 3) similar to that in the SKBR3 parent cells (Lane 1). Treatment of the cells with 1 μM PMA under serum-free conditions for 16 hours resulted in a marked matriptase down-regulation in the SKBR3 parent and the SKBR3/CC-SCR cells (Figure 7, Lanes 2 and 4). The PMA-induced matriptase down-regulation is at the protein level with no matriptase mRNA down-regulation as determined by quantitative RT-PCR (data not shown). The PMA-induced matriptase down-regulation was associated with a reduction of the full-length Her2 in the parent SKBR3 and the SKBR3/CC-SCR cells, by 25% and 11%, respectively. In the SKBR3/CC-ST14 cells, the PMA-induced full-length Her2 reduction was further enhanced, to 44%; in association with the total loss of matriptase expression (Figure 7, Lane 6). The sustained PMA exposure also abolished PKCα protein in all three cell types (Figure 7, Lanes 2, 4, and 6).

## DISCUSSION

In this study we employed a co-expression system to bring membrane-associated serine proteases and their candidate RTK substrates together in a “cellular test tube” to determine the specific enzyme-substrate relationships. Unlike testing the substrates for soluble enzymes such as trypsin, wherein a pure enzyme and a pure substrate can be mixed together for analysis in a test tube; the testing of the candidate substrates for the membrane-associated extracellular serine proteases requires the membrane topological context. In our previous study [2] matriptase was shown to generate 110-kDa and 135-kDa EGFR CTFs when the receptor and the protease were co-expressed in the cellular test tube, but these CTFs were not seen when the purified receptor and the pure enzyme out of the membrane context were mixed together. Instead, most likely biologically irrelevant CTFs at 76 and 63 kDa were produced when matriptase can now access the receptor's intracellular portions, which are not accessible if the protease and the receptor are properly located on the cellular membrane. One caveat of using the “cellular test tube” to analyze the membrane proteases’ substrates is that there may always be an intermediate enzyme or factor between any specific pairs of protease and substrate candidate being tested. With this caveat in mind, we can state that matriptase and hepsin are Her2 ECD-cleaving proteases by looking at our current data. The case for matriptase having a direct proteolytic cleaving role on Her2 is further strengthened with the absence of site-specific CTFs in the experiments involving the R558A and R599A Her2 mutants. The sites at Arg558 and Arg599 conform to the best matriptase substrate [10] thus the failure for matriptase to cleave the Arg/Ala mutants would be a strong indication that matriptase is performing the cutting. There are indeed minor CTF species of the cleaved Her2 in the matriptase-expressing cells, as shown in Figure 4A (Lanes 2, 4, and 6) and also in Figure 6 (Lane 2). These may well be CTFs generated by other proteases that are activated by matriptase.

Proteolytic shedding of an RTK's ECD is a mechanism of regulating cell signaling mediated by the RTK. Metalloproteases of the ADAM (A Disintegrin And Metalloprotease) family have long been recognized to perform this function. For example, ADAM10 is a major sheddase of the Her2 ECD in Her2-over-expressing breast cancer cells [19]. Unidentified serine proteases expressed in human breast cancer cell lines SKBR3 and BT474 had been implicated for a role in Her2 ECD shedding upon protein kinase C (PKC) activation, producing CTFs capable of activating downstream signaling [20]. Our laboratory was the first to identify matriptase as a serine protease capable of shedding the ECD from an RTK, the Her1/ErbB1/EGFR [1]. We have since demonstrated that another TTSP, hepsin, was also capable of shedding the EGFR ECD [2]. The proteolytic events that we showed on EGFR required co-expression of the ECD-shedding serine protease and the receptor via a co-transfection of their cDNAs at the same time [1]. Expressing exogenous EGFR via transfection in a cell line such as the BPH-1 that expresses an abundance of matriptase (unpublished data) did not produce the matriptase-mediated cleavages [1]. The EGFR cleavages by matriptase were independent of receptor phosphorylation [1]. In the current study, the Her2 cleavages by matriptase at Arg558 and Arg599 were also observed in co-transfection experiments using the HEK293 cells. The Her2 ECD cleavages by matriptase however, occur on phosphorylated Her2 (at tyrosines 1221/1222) but not unphosphorylated Her2 (Figure 5B). Unlike the EGFR which requires ligand binding to induce activation of the intracellular tyrosine kinase domain and receptor phosphorylation, Her2 is in a perpetually open form and does not have a ligand. Her2 clusters on the plasma membrane in cells that over-express the receptor and beyond a density threshold will homo-dimerize to phosphorylate [16]. Her2 lacks internalization signals and can only undergo endocytosis at the basal level, while it recycles quickly to the plasma membrane when internalized to maintain a high level of membrane distribution [21]. These unique structural features of Her2 suggest that the phosphorylated Her2 is present on the plasma membrane and thus the Her2 ECD cleavages by matriptase occur also on the plasma membrane, not co-translationally.

We selected Her2 and matriptase along with the reversible matriptase inhibitor HAI-1 to study further in human breast cancer cells because there is a significant clinical relevance of matriptase in relation to Her2 in breast cancer. In a study involving 107 primary breast tumors, matriptase expression was found to correlate strongly with Her2 expression at the protein level (Spearman's Rho 0.57) [22]. The study further determined that among 16 human breast cancer cell lines tested, the Her2-amplified SKBR3 and BT-474 had high matriptase expression, confirming the results of a previous study [15]. In a study involving 377 invasive breast cancers, negative or low matriptase expression was shown to be associated with poor survival [23]. Re-expression of matriptase in the matriptase-negative human breast cancer cell line MDA-MB-231 inhibited tumor xenograft growth *in vivo* albeit only modestly [22]. On the other hand, silencing matriptase expression in mice delayed breast tumor formation and inhibited tumor growth via down-regulating the c-MET oncogenic pathway [24]. An earlier study involving 330 cases of node-negative breast cancers revealed a rate of 45% (148/329) as expressing high levels of matriptase and 55% (181/329) as expressing low levels [25]. The reversible Kunitz-type major matriptase inhibitor HAI-1 is functionally relevant in breast cancer cells in its matriptase inhibition role [26]. In the study by Kang et al. [25] however, only 18% (18/316) of node-negative breast cancers expressed high levels of HAI-1 while 82% (260/316) presented low HAI-1 expression. Based on the expression of matriptase, HAI-1, and Her2 in breast cancers evaluated in these previous studies, a case can be made for examining the interplays of Her2, matriptase, and HAI-1 in breast cancer. Human breast cancer cell lines available for mechanistic studies typically express high levels of matriptase, HAI-1, and Her2, such as the SKBR3 and BT-474, or, none of the three molecules, such as the MDA-MB-231. Derivative cell lines of the MDA-MB-231 genetically engineered to over-express Her2 have previously been applied to clinically relevant anti-Her2 drug resistance research [17]. Thus, our 231H2-TRM cell lines based on the MDA-MB-231 with Her2-over-expression and the inducible matriptase expression; or additionally a constitutive HAI-1 expression are potentially relevant as a model to investigate the impact of Her2 ECD cleavage by matriptase on anti-Her2 drugs. The inducible matriptase expression allowed us to determine if a stably expressed Her2 can be cleaved by the protease, and we had observed exactly this phenotypic outcome; whereas the Her2 mutant without the Arg558 and Arg599 matriptase cleavage sites was not affected (Figure 6). The constitutive HAI-1 expression completely abolished the matriptase cleavages of Her2, so these cleavages in this breast cancer cell line at least require matriptase and most likely are by matriptase as the sites at Arg558 and Arg599 conform to the optimal matriptase substrate [10].

The SKBR3 and the BT-474 human breast cancer cell lines are Her2-amplified and express a high abundance of this receptor at the protein level. At the same time, these two cell lines also express a high abundance of matriptase at the protein level [15], as well as a high level of HAI-1, which can effectively inhibit matriptase cleavage of Her2 in a re-expression context in the MDA-MB-231 cells, as we have shown in this study (Figure 6). The co-expression of the highly efficient matriptase inhibitor HAI-1 can help explain that under normal conditions these Her2-amplified human breast cancer cells do not present matriptase cleavages of Her2. Alternatively, differential membrane localization may be a potential mechanism by which an RTK and its ECD-shedding TTSP can be present in the same cell without or with only minimal active ECD shedding. It has been reported that PKC activation via PMA stimulation results in matriptase translocation to cell-cell contacts whereat matriptase can cleave the Tie2 ECD in the same or the neighboring cells [4]. In the absence of PKC activation matriptase is dispersed throughout the plasma membrane whereas PKC activation is required for matriptase translocation to cell-cell contacts [27]. TMPRSS6 (matriptase-2), whether expressed exogenously in the HEK293 cells via transfection or endogenously in the HepG2 cells or primary hepatocytes, undergoes constitutive internalization via a dynamin-dependent pathway [28], reducing its interactions with and actions on its physiological substrate hemojuvelin. Cells expressing a mutant TMPRSS6 incapable of endocytosis retained the protease at the cell surface and had a sustained hemojuvelin substrate cleavage. Considering that PKC activation can inhibit dynamin-dependent endocytosis [29], a PKC activation event may be the physiologically or pathologically relevant scenario under which RTK ECD-shedding TTSPs such as matriptase are called into action to proteolytically modify the receptors co-expressed in the same cell or expressed in neighboring cells according to the cell's signaling requirements. The lesson from the examples of cellular event-conditional cleavages of Tie2 by matriptase or of hemojuvelin by TMPRSS6 is that short of a proper stimulus to bring the enzyme and the substrate in the exact cellular context required for cleavage the cleavage would not take place, or not at a level to allow for detection.

In this study we investigated matriptase and Her2 changes in the parent SKBR3 cells, and SKBR3 sublines with the matriptase gene knockout or harboring a scrambled sgRNA CRISPR/Cas9, upon exposure to 1 μM PMA under serum-free conditions for a sustained period of 16 hours. Our data would suggest that matriptase has a mechanistic role in Her2 expression and its absence is associated with Her2 down-regulation, an event manifesting more prominently during long-term exposure to PMA (Figure 7). In other words, the presence of matriptase would prevent the PMA-induced Her2 turnover. This role is in agreement with the previously reported coordinated expression of matriptase and Her2 in human breast cancers and cell lines [22], and with the poor clinical outcome associated with low or no matriptase expression [23]. The role of PKC in Her2 turnover and recycling has been investigated to some extent but the overall picture remains complex. PKCα and PKCδ have been shown to promote Her2 entry into the endocytic recycling compartment (ERC) from the cell surface in the SKBR3 human breast cancer cells [30]. In that study, treating the SKBR3 cells with 0.1 μM PMA for 8 hours was not accompanied with a reduction of total Her2. PKCα was also reported to have a role in recycling Her2 to the cell surface in Her2-overexpressing but unamplified breast cancer cells; whereas reducing PKCα by 50% via siRNA resulted in a 40% reduction of total Her2 [31]. Curiously, in a much earlier study of Her2 turnover and recycling, a chimeric Her2 carrying the cytoplasmic domain of Her2 but the extracellular domain of EGFR, the Her1-2, was degraded upon treatment with 1 μM PMA for 4 hours when co-expressed with PKCα in the HEK293 cells [32]. Long-term treatment of cells with PMA however, at a range of doses and times, 0.1-1 μM PMA for 12-24 hours, was reported to down-regulate the various forms of PKC in various cell types [33, 34, 35, 36]. In the SKBR3 cells treated with 1 μM PMA for 16 hours we chose PKCα as a representative isoform for evaluation, and observed a complete abolishment of PKCα (Figure 7). This observation is in agreement with the previously reported PKCα changes in response to a sustained exposure to PMA. It is probable that the PMA abolishment of PKCα takes away a mechanism to recycle Her2, which would then enter a lysosomal pathway following the basal level internalization. Over time, the total cellular level of Her2 would decrease, whereas matriptase's presence seemed to slow down this process. Matriptase has been shown to activate PKCζ which then mediates claudin-2 protein turnover without affecting the claudin-2 mRNA levels [37]. Silencing of matriptase expression by siRNA resulted in accumulation of claudin-2, a phenotype that was reproduced by inhibiting PKCζ. The matriptase - PKCζ - claudin-2 pathway was proposed to be a mechanism by which matriptase regulates epithelial barrier formation and permeability. There is undoubtedly an interface between matriptase and PKC activities, and one between PKC and protein turnover and recycling. The fate of the specific protein that matriptase affects, such as that of claudin-2 and Her2, may depend on the specific cell type with a specific profile of PKC isoforms.

Cell-surface specific interaction of Herceptin with Her2 has recently been established [38], supporting the confirmatory results in pulling down the full-length Her2 in our cell-surface binding experiments. More important, our intent was to show that this specific interaction would be abolished for CTF-Cys600 due to a complete loss of the Herceptin binding sites in the Her2 ECD, and our results support this hypothesis. Shedding of the Her2 ECD by metalloproteases or alternative translational initiation can produce CTFs no longer recognizable by the monoclonal antibody cancer drug Herceptin (reviewed in [39]), presenting challenges to treating Her2-positive breast cancers and also mechanisms for Herceptin resistance. One form of the matriptase-cleaved Her2, CTF-Cys600 (cleaved at Arg599) can be expected to have a similar effect or impact on Herceptin response as some of the previously described Her2 CTFs [39], because CTF-Cys600 is not recognizable by Herceptin. It is reasonable to suggest that the matriptase-mediated Her2 ECD shedding be considered as a potential new mechanism underlying Herceptin resistance, de novo or acquired.

The wild-type protease-active TTSPs were fully expected to show activities in shedding of the RTK ECDs, as we had previously observed for EGFR and have shown in the current study for additional RTKs. But the protease-dead matriptase (Mat-Mut) was observed to have an effect of significantly reducing the expression level of some RTKs, such as Her3, Her4, and the two PDGFRs but leaving no detectable levels of any CTFs. We had previously shown that over-expression of a protease-dead prostasin in the human prostate cancer cell line PC-3 resulted in a very robust induction of matriptase expression, which was associated with a reduction of the EGFR level [40]. The protease-dead matriptase may very well have an induction effect on a downstream protease or even a protease-independent mechanism to result in a reduction of the RTK levels. Identifying the downstream mechanism will be pursued in our continued research.

## CONCLUSIONS

We were able to show that the TTSPs matriptase and hepsin exhibit ECD-shedding activities toward members of the RTK family receptors previously not examined. These include three members of the Her/ErbB subfamily, two members of the insulin receptor subfamily, two members of the PDGFR subfamily and one member of the nerve growth factor receptor subfamily. The TTSP TMPRSS6 has a more limited range of RTK substrates, cleaving only Her4, INSR, and PDGFR α and β. Prostasin, a GPI-anchored serine protease was only weakly active toward Her3 and PDGFRα but was unable to cleave any other RTKs tested. At least for Her2, the new information reported in this study on matriptase cleavage and the interactions of the CTFs with the monoclonal antibody cancer drug Herceptin may have a practical application beyond our initial intent of testing for test tube substrate cleavages in the cellular membrane-associated context. In human breast cancer cells matriptase has a mechanistic role in regulating Her2 expression. The significance for all other RTK cleavages by the TTSPs and prostasin will require much broader and deeper levels of continued research investigation to establish by all parties with interest in these receptors and proteases.

## MATERIALS AND METHODS

### Cell lines and cultures

The human embryonic kidney epithelial cell line HEK293 expressing the Epstein-Barr nuclear antigen 1, HEK293-EBNA (HEK293 hereon) was obtained from Invitrogen (Carlsbad, CA) and cultured in DMEM supplemented with 10% fetal bovine serum (FBS). The human M17 neuroblastoma cells (kind gift from Dr. Sic-Lung Chan, US Food and Drug Administration, Silver Spring, MD) were cultured in OPTI-MEM I (Invitrogen) supplemented with 10% FBS. The source and culturing conditions for the human breast cancer cell line MDA-MB-231 and JIMT-1 were described previously [1, 41]. The human breast cancer cell lines SK-BR-3(SKBR3), BT-474, and MCF-7 were obtained from the American Type Culture Collection (Manassas, VA), and cultured in the same conditions as those for MDA-MB-231 and JIMT-1.

### Plasmids coding for proteases and RTKs

The cDNA plasmids coding for human matriptase, prostasin, and hepsin and their protease-dead mutants were described previously [2]. The cDNAs coding for the mouse TMPRSS6 (Addgene Plasmid 18789, Bruce Beutler [5]) and a protease-dead mutant mouse TMPRSS6 (S762A, Addgene Plasmid 18791, Bruce Beutler [5]) were obtained from Addgene (Cambridge, MA), along with the cDNAs coding for human insulin receptor (INSR, Addgene Plasmid 24049, Frederick Stanley [6]), insulin-like growth factor I receptor (IGF-1R, Addgene Plasmid 11212, Ronald Kahn [7]), the platelet-derived growth factor receptors (PDGFRs) α (Addgene Plasmid 23892, William Hahn [8]) and β (Addgene Plasmid 23893, William Hahn [8]), and nerve growth factor receptor A (TrkA) (Addgene Plasmid 23891, William Hahn [8]). The cDNA coding for human Her3/ErbB3 (SC118918) was purchased from OriGene Technologies (Rockville, MD). The cDNAs coding for human Her2/neu/ErbB2 and Her4/ErbB4 were amplified from the total RNA of human breast cancer cell line BT-474 (American Type Culture Collection, Manassas, VA) following reverse transcription using the SuperScript III reverse transcriptase (Invitrogen). The complete coding sequence for each RTK was amplified by polymerase chain reaction (PCR) using the Phusion DNA polymerase (ThermoFisher, Pittsburgh, PA) and subcloned to generate a cDNA coding for a carboxyl-terminally hemagglutinin (HA)-tagged RTK according to the methods described previously [1].

A cDNA coding for a glycosylphosphatidylinositol (GPI)-anchored matriptase was generated by inserting the PCR-amplified matriptase protease domain coding sequence (codons Gly596-Val855, NM_021978) into the backbone of the human prostasin cDNA with the prostasin protease domain coding sequence deleted (codons Ala51-Leu288, NM_002773). A cDNA coding for a GPI-anchored protease-dead matriptase mutant (S805A) was constructed likewise but using the previously described matriptase mutant cDNA [1] as the template for PCR amplification. Her2 ECD mutants with alanine substitutions for arginines at positions 558 and 599 were generated by using the QuickChange Site-Directed Mutagenesis Kit (Agilent Technologies, Inc., Santa Clara, CA). Her2 ECD deletion mutants Δ23-558, Δ23-599, and Δ23-647 were generated by PCR using the Phusion DNA polymerase.

### Protease cleavage assay

HEK293 cells were seeded at 4 × 10^5^ per well in 12-well plates coated with poly-L-lysine at 18 hours before transfection. The plasmid containing a cDNA coding for human matriptase, protease-dead matriptase mutant (S805A), hepsin, protease-dead hepsin mutant (S352A), prostasin, GPI-anchored matriptase, GPI-anchored protease-dead matriptase mutant, mouse TMPRSS6, or mouse TMPRSS6 protease-dead mutant (S762A), at 0.4 μg, was co-transfected with 0.6 μg of the plasmid containing a cDNA coding for an HA-tagged RTK using the Lipofectamine 2000 reagent according to the supplier's protocol (Invitrogen). At 24 hours after the transfection, cells were lysed in RIPA buffer and the lysate was analyzed by SDS-PAGE and western blotting for various target proteins or fragments using an anti-HA antibody as described previously [1].

HEK293 cells transfected with Her2(HA), matriptase, or Her2(HA) and matriptase, as above, were used along the human breast cancer cell lines SKBR3, BT-474, JIMT-1, MCF-7, and MDA-MB-231 for western blot analysis of Her2 (using an antibody against the native Her2, 29D8, Cell Signaling Technology, Danvers, MA) and matriptase to evaluate the relative expression levels of the receptor and the protease. The HEK293 cells transfected with Her2(HA), matriptase, or Her2(HA) and matriptase were also used for western blot analysis of phosphorylated Her2 (at tyrosine 1221/1222) with an anti-phospho-Her2 antibody (6B12, Cell Signaling Technology). A GAPDH western blot was performed as described previously [1], on the same membrane, as a loading control.

### Immunoprecipitation (IP) with Herceptin

The M17 neuroblastoma cells were seeded in 12-well plates and transfected using the procedures described above. At 18 hours past transfection the cells were placed under fresh culture medium with 150 μg/ml Herceptin (generously provided by Genentech, South San Francisco, CA). After 24 hours of Herceptin incubation the cells were washed twice with ice-cold phosphate-buffered saline (PBS) and lysed in a lysis buffer (20 mM Tris, pH 8.0, 150 mM NaCl, and 1% NP-40). Forty micrograms of lysate per sample were then mixed with 5 μl of agarose beads bearing goat anti-human IgG (Fc-specific) (Sigma-Aldrich, St. Louis, MO) for an hour at 4°C. Before the pull-down assays the agarose beads were used for preclearing the cell lysates produced with duplicate samples without the addition of Herceptin for binding to the cells. The full-length Her2 or CTF-His559 was not pulled down by the beads alone in the preclearing (data not shown). The beads were then washed with the lysis buffer for 4 times, and subjected to SDS-PAGE and western blot analysis using an anti-HA antibody as described [1]. The post-IP lysate for each sample was also analyzed.

### Establishing a Her2-over-expressing MDA-MB-231 derivative cell line, the 231H2

The human Her2(HA) cDNA in the pcDNA3 vector (Invitrogen) described above was transfected in the MDA-MB-231 cells using Lipofectamine 2000. The transfected cells were cultured in 100-mm dishes under G418 selection (500 μg/ml) until visible colonies formed. Colonies were picked using a micropipette tip with trypsin and cultured in individual dishes. Her2 expression levels in the colonies were determined by SDS-PAGE and western blotting as described above. A colony with ascertained over-expression of Her2 was designated 231H2. An MDA-MB-231 derivative cell line over-expressing a Her2(HA) mutant with alanine replacements at Arg558 and Arg599, the 231R2A, was established likewise using the mutant Her2(HA) cDNA carrying the mutated codons.

### Establishing 231H2 and 231R2A derivative cell lines expressing a tet-induced protease

The cDNA coding for matriptase was subcloned into the pLenti4/TO/V5-DEST vector (Invitrogen) to produce lentiviruses. The 231H2 and 231R2A cells were transduced with a lentivirus expressing the tetracycline repressor (TR) in the pLenti6/TR vector (Invitrogen) to produce the derivative lines 231H2-TR and 231R2A-TR, respectively. Lentiviruses expressing tet-regulated matriptase in the pLenti4/TO/V5-DEST vector were used to transduce the 231H2-TR or the 231R2A-TR cells to establish two corresponding lines with tet-induced matriptase expression on the background of Her2 or R2A over-expression, the 231H2-TRM and the 231R2A-TRM. A full-length cDNA encoding the human hepatocyte growth factor activator inhibitor-1 (HAI-1) [15] (a gift from Dr. Chen-yong Lin, Georgetown University, Washington, D.C.) was subcloned into the pLVX-Puro vector (Clontech Laboratories, Inc., Mountain View, CA) to produce lentiviruses used to transduce the 231H2-TRM cells. The resulting cell line, 231H2-TRM-H1 expresses constitutive human HAI-1 over the background of constitutive Her2(HA) and tet-inducible matriptase expression. Induction of matriptase expression in the TRM cells was performed by adding tetracycline (1 μg/ml) to the culture medium. Cells were kept in the tetracycline-containing medium for 24 hours before lysis and western blot analysis. The HAI-1 antibody was purchased from Santa Cruz Biotechnology (H-180, Santa Cruz, CA).

### Establishing SKBR3 derivative cell lines with matriptase gene knockout

Lentiviral vectors containing an ST14 sgRNA CRISPR/Cas9 All-in-One matriptase (ST14) gene targeting system or a scrambled sgRNA were obtained from Applied Biological Materials, Inc. (Richmond, BC, Canada). The target sequence of the matriptase (ST14) sgRNA is: 5′-CTTTGTGGTCACCTCAG-3′; and that of the scrambled sgRNA is: 5′-GCACTCACA TCGCTACATCA-3′. Lentiviruses expressing the sgRNA CRISPR/Cas9 were used to transduce the SKBR3 cells to produce the derivative cell lines SKBR3/CC-SCR (scrambled) and SKBR3/CC-ST14 (matriptase targeting). The SKBR3 parent, SKBR3/CC-SCR, and SKBR3/CC-ST14 cells were seeded in 12-well plates at 500,000 cells per well and cultured overnight. The cells were then placed under serum-free EMEM with either 1 μM PMA or an equal amount of the solvent (DMSO, dimethyl sulfoxide) and cultured for another 16 hours before lysis and western blot analysis. The PKCα antibody was purchased from Transduction Laboratories (Lexington, KY).

## SUPPLEMENTARY MATERIALS FIGURES


