# Supplementary material for: Proteolytic cleavages in the extracellular domain of receptor tyrosine kinases by membrane-associated serine proteases
Source: Oncotarget. 2017 Apr 10;8(34):56490–505. doi: 10.18632/oncotarget.17009 (PMC5593577; doi:10.18632/oncotarget.17009)
Supplement: Supplementary file 1 [file oncotarget-08-56490-s001.pdf]

Proteolytic cleavages in the extracellular domain of receptor tyrosine kinases by membrane-associated serine proteases

SUPPLEMENTARY MATERIALS

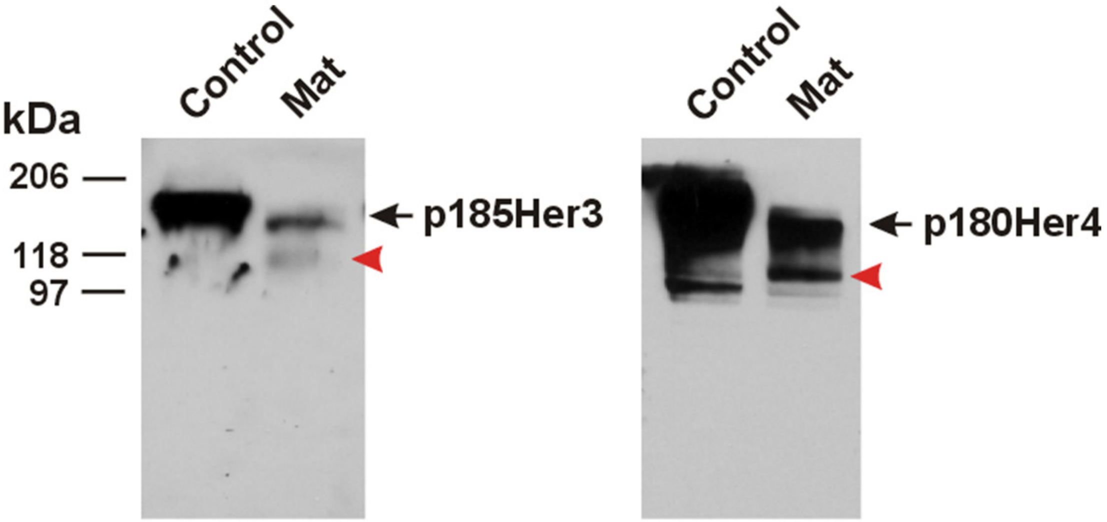

Supplementary Figure 1: Her3 and Her4 cleavages by matriptase. The images were obtained by performing a longer exposure of the membranes used for generating the images in Figure 1B and Figure 1C to show the matriptase cleavage product of Her3 and Her4 in the left and right panels, respectively. The full-length receptors are indicated by the black arrowheads and the CTFs are indicated by the red arrowheads.
